# Quality of Life Outcomes of Antiretroviral Treatment for HIV/AIDS Patients in Vietnam

**DOI:** 10.1371/journal.pone.0041062

**Published:** 2012-07-20

**Authors:** Bach Xuan Tran

**Affiliations:** 1 School of Public Health, University of Alberta, Edmonton, Canada; 2 Institute for Preventive Medicine and Public Health, Hanoi Medical University, Hanoi, Vietnam; Johns Hopkins Bloomberg School of Public Health, United States of America

## Abstract

**Objective:**

This study assessed health-related quality of life (HRQOL) and its related factors in HIV/AIDS patients taking antiretroviral treatment (ART) in Vietnam.

**Methods:**

A cross-sectional study was conducted with 1016 patients (36.2% women, mean age = 35.4) in three epicenters of Vietnam, including Hanoi, Hai Phong, and Ho Chi Minh City. HRQOL was assessed using the Vietnamese version of the WHOQOL-HIV BREF. Factor analysis classified measure items into six HRQOL dimensions, namely Physical, Morbidity, Social, Spirituality, Performance, and Environment. Tobit censored regression models were applied to determine associations of patient’s characteristics and HRQOL domain scores.

**Results:**

Internal consistency reliability of the six domains ranged from 0.69 to 0.89. The WHOQOL-HIV BREF had a good discriminative validity with patient’s disease stages, CD4 cell counts, and duration of ART. In a band score of (4, 20), six domains were moderate; “Environment” had the highest score (13.8±2.8), and “Social” had the lowest score (11.2±3.3). Worse HRQOL were observed in patients at provincial and district clinics. Those patients who were male, had higher educational attainment, and are employed, reported better HRQOL. In reduced regression models, poorer HRQOL was found in patients who had advanced HIV infection and had CD4 cell count <200 cells/mL. Patients reported significantly poorer Physical and Social in the 1^st^ year ART, but moderately better Performance, Morbidity, Spirituality, and Environment from the 2^nd^ year ART, compared to those not-yet-on ART.

**Conclusion:**

Strengthening the quality of ART services at the provincial and district levels, gender-specific impact mitigation, and early treatment supports are recommended for further expansion of ART services in Vietnam. Regular assessments of HRQOL may provide important indicators for monitoring and evaluating HIV/AIDS services.

## Introduction

Globally, scaling up antiretroviral treatment (ART) has considerably relieved the socioeconomic and health burden of HIV/AIDS epidemics [1]–[3]. HIV/AIDS patients who are receiving ART could achieve suppressed viral loads, improved immune and physical functions, and reduced opportunistic infections and co-morbidities. In addition, they might continue to be productive, more socially inclusive, and have better quality of life [4], [5]. Since ART is life-long, monitoring ART outcomes is necessary to ensure quality and efficiency of health system.

Health-related quality of life (HRQOL) is an important indicator to assess the impact and quality of health care system [6]. It reflects the patient perspective on various aspects of health, ranging from symptomatic to more complex concepts, such as social functioning or spirituality [6]. In HIV/AIDS research, there has been a growing body of literature on HQOL measurements [4], [5], [7], [8]. HRQOL was associated with clinically important outcomes of ART, including treatment adherence, immunological and virological changes [9]–[11]. Moreover, HRQOL could reflect non-medical aspects of living with HIV/AIDS [12], [13]. Poorer HRQOL was observed in patients perceived less psychosocial supports [4], [12], and experienced depressive symptoms [12], [14]. Consequently, assessments of HRQOL may inform the design and evaluation of interventions, identify the need for health services improvements, and monitor changes in health status of HIV/AIDS patients [4].

Vietnam has experienced a rapid growing HIV epidemic in Asia [15], [16] with high prevalence of HIV seen in most-at-risk groups, including injecting drug users (13.4%), female sex workers (3%), and men who have sex with men (16.7%) [17]. Since 2006, ART services have rapidly been scaled up in Vietnam [18]–[20]. By 2010, 318 ART clinics have been established, and more than 50% ART-eligible patients have received treatment [21]. In the rapid expansion of ART coverage, understanding its outcomes and identifying strategies for improving its service quality are urgently needed. This study assessed HRQOL of HIV/AIDS patients taking ART, and determined factors associated with HRQOL outcomes of ART services in Vietnam.

## Methods

### Ethics Statement

This is a part of the research project on “Cost and cost-effectiveness of HIV/AIDS care and treatment policy options in Vietnam”. The use of data was approved by the Authority of HIV/AIDS Control, Ministry of Health of Vietnam. Ethical approval was granted by the University of Alberta’s Health Research Ethics Board. Written informed consent was obtained from all participants after a clear explanation of the survey. Respondents were able to refuse participating in and withdrawing the interview at any time, and this did not affect continuation of health care services for the participants. Confidentiality was assured using codes of patient’s information, and secured storage was prepared for both paper questionnaires and electronic dataset.

### Study Settings

We conducted a cross-sectional study in 3 epicenters of Vietnam, namely the 2012 Vietnam HIV Services Users Survey (HSUS). Three metropolitan areas, including Ha Noi, Hai Phong, and Ho Chi Minh City, were selected to represent different geographical areas of the largest HIV epidemics in Vietnam. Ha Noi is the capital city with a population of 6 million, of those, approximately 18,000 people living with HIV. Hai Phong is a port city in northern Vietnam with 1.8 million citizens, of those, 6930 reported HIV-positive. Ho Chi Minh City is the largest southern metropolitan center with the largest HIV-positive population of 46,507 cases over 8 million residents [22], [23].

### Study Design and Participant Recruitment

Seven hospitals across levels of health system governance (central, provincial and district) were purposively selected for the study where 1016 patients were interviewed (17% of total sample frame). Sample size was determined for each hospital on a proportional basis. It included 201 patients (20%) at the National Hospital for Tropical Diseases, 406 patients (40%) at 3 provincial hospitals (Dong Da, Viet Tiep, and Ho Chi Minh City Tropical Diseases Hospital), and 409 patients (40%) at 3 district health centers (Tu Liem, Le Chan, and Binh Tan). Patients with HIV/AIDS were randomly selected and invited to participate during their clinics visits.

### Measures and Instrument

We interviewed patients using a structured questionnaire to collect information about their socioeconomic and clinical status. The socioeconomic characteristics included age, gender, marital status, education level, employment, and income. Household’s monthly income was self-reported by summing up all sources of income from each member over the past year; and then stratified into five quintiles. Clinical information included HIV/AIDS stages, CD4 cell count, history of drug use, and duration of ART. Patients were also asked to self-fill in a paper-based HRQOL questionnaire. For those patients who were severely ill or experienced any difficulty in filling the form, we interviewed them instead.

*The World Health Organization Quality of life - HIV brief instrument* (WHOQOL-HIV BREF) was employed for measuring HRQOL of HIV/AIDS patients. This is a multidimensional profile, which includes 31 items covering 6 domains: Physical (4 items), Psychological (5 items), Level of Independence (4 items), Social Relationships (4 items), Environment (8 items), and Spirituality (4 items); and 2 other general items (Overall HRQOL and General Health) [24], [25]. The respondents answered each question using a 5-item Likert scale. For example, patients were asked to think about their life in the last 2 weeks in the question: “To what extent are you bothered by people blaming you for your HIV status?” Answering options would be: “1- Not at all; 2- A little; 3- A moderate amount; 4- Very much; and 5- An extreme amount”. The scoring system reversed score in 6 questions to make higher scores generally indicated better HRQOL [26], [27]. The summary domain and total HRQOL scores were calculated using the scoring method developed by WHOQOLHIV group [24]. Responded values of each item contributed equally to the corresponding domain scores. We multiplied the average scores of all items in each domain by four to convert domains’ scores to the range of [4]; [20], making it comparable with scores derived from the WHOQOL-100. The Vietnamese version of WHOQOL-HIV BREF has been developed and presented elsewhere [5].

### Statistical Analysis

Socioeconomics and HIV-related characteristics of respondents were presented in proportions and means. ANOVA test was used to examine the differences between average HRQOL domain scores.

*Psychometric properties of the HRQOL measurement*: Internal consistency reliability of measurement was estimated using Cronbach’s alpha. *Exploratory factor analysis (EFA)* was applied to examine the construct of HRQOL measurement using the WHOQOL-HIV BREF. EFA identified the number of factors, grouped items into determined factors should they share sufficient variation to justify their existence as a construct to be measured. Six factors were extracted by the principle component analysis at an eigenvalue of 1.0, which was the threshold, defined using the scree test, where the eigenvalue curve flattened out. Orthogonal Varimax rotation with Kaisers’ normalization was used to reclassify the measure items in order to increase the interpretability of these factors. The cut off point for factor loadings was set at 0.40. We had two cross-loadings, thus, associated items were assigned it to the corresponding domain based on the nature of the questions and the overarching dimension. We examined the discriminative validity of measurement by testing an ‘a priori’ hypothesis that the WHOQOL-HIV BREF was capable to distinguish patients at different HIV/AIDS stages and immunological status. Domains scores were correlated with patients’ general quality of life and satisfaction with health status to examine the convergent validity of the measurement. Finally, we examined the longitudinal validity of the HRQOL measurement with the length of taking ART.

*Multivariable linear regression* was performed to determine factors associated with HRQOL domains scores. Since HRQOL domains scores raged at [4]; [20], thus, they were left- and right censored. Censoring from above and below the HRQOL domain scores did not allow us to measure exactly the values which were higher or lower than the range thresholds. Therefore, we employed censored regression models or Tobit models to estimate linear relationships patients’ characteristics and HRQOL domain scores in regression analysis [28]. We applied a stepwise forward model building strategy which selected variables based on the log-likelihood ratio test at a p-value <0.1, and excluded variables at p-values >0.2 [29].

## Results

### Socio-demographic and Clinical Characteristics of Respondents

Of 1016 patients interviewed, 63.8% were male, average age was 35.4 (SD = 7.0), 45% had high school education and above, and 20.4% had stable jobs. Over one third of respondents were in AIDS stage (37.6%), half of them were symptomatic HIV stage (50%) and only 12.4% were asymptomatic. There were 88.8% patients taking ART, among those, 55.3% have been treated for more than 2 years. The majority of patients remained low CD4 cell counts; 86.4% had CD4 less than 500 cells/mL (Table 1).

**Table 1:** Patient characteristics by level of health system.

				Performance		Physical		Morbidity		Social		Spirituality		Environment	
	N	%		Mean	SD	Mean	SD	Mean	SD	Mean	SD	Mean	SD	Mean	SD
**All**	**1016**	**100.0**		12.6	2.3	13.2	3.1	12.7	3.5	11.2	3.3	12.6	2.9	13.8	2.8
**Level of Health System**															
Central	201	19.8		13.1***	2.1	14.0***	2.8	12.9**	3.2	12.4***	3.2	13.1***	2.5	14.3***	2.5
Provincial	406	40.0		12.3	2.4	12.6	3.3	12.4	3.7	10.6	3.2	12.2	3.0	13.4	3.0
District	409	40.3		12.7	2.3	13.5	2.9	13.1	3.5	11.2	3.4	12.7	2.9	13.9	2.8
**Age**															
< = 35 yrs old	584	57.5		12.6	2.3	13.2	2.9	12.7	3.5	11.0	3.3	12.7	2.8	13.8	2.8
>35 yrs old	432	42.5		12.6	2.3	13.3	3.3	12.7	3.5	11.4	3.4	12.5	3.1	13.8	2.9
**Gender**															
Male	648	63.8		12.6	2.4	13.2	3.1	12.7	3.6	11.3	3.4	12.6	2.9	13.8	2.9
Female	368	36.2		12.6	2.2	13.3	3.0	12.8	3.5	11.0	3.3	12.5	2.9	13.7	2.7
**Education**															
Below high school	556	54.7		12.4***	2.3	12.9***	3.2	12.6	3.7	10.6***	3.3	12.3***	2.9	13.5***	2.9
High school and above	460	45.3		12.9	2.3	13.6	3.0	12.9	3.3	11.9	3.3	12.9	2.9	14.1	2.7
**Marital status**															
Single	142	14.0		12.4	2.6	12.7**	3.7	12.4	3.9	10.8	3.4	12.0**	3.3	13.5	3.3
Live with spouse/partner	650	64.0		12.7	2.2	13.4	2.9	12.7	3.4	11.3	3.3	12.7	2.8	13.9	2.7
Widow(er), Divorced	224	22.1		12.4	2.4	13.1	3.2	13.0	3.6	11.1	3.4	12.6	3.0	13.8	2.8
**Employment**															
Unemployed	181	17.8		12.3	2.5	12.4***	3.6	12.0**	3.7	10.8**	3.5	12.3	3.0	13.5	3.1
Free lancer	534	52.6		12.6	2.3	13.4	2.9	12.9	3.5	11.1	3.4	12.6	2.9	13.8	2.8
Stable jobs	207	20.4		12.9	2.0	13.5	2.8	12.9	3.3	11.7	3.1	12.8	2.8	14.0	2.6
Others	94	9.3		12.8	2.3	13.2	3.3	12.9	3.6	10.9	2.9	12.4	3.0	13.9	3.0
**Income quintiles**															
Poorest	184	20.0		12.5	2.3	13.1*	3.1	12.8*	3.3	11.1*	3.2	12.6**	2.9	14.0	2.7
Poor	166	18.0		12.3	2.4	12.8	3.3	12.3	3.4	10.7	3.2	11.9	2.6	13.4	3.0
Medium	192	20.9		12.7	2.2	13.2	2.8	12.9	3.6	11.2	3.4	12.4	2.9	13.7	2.7
Rich	194	21.1		12.6	2.2	13.4	3.0	12.8	3.5	11.3	3.2	12.5	2.9	13.8	2.9
Richest	184	20.0		12.9	2.3	13.7	3.0	13.0	3.8	11.5	3.5	13.0	3.1	13.9	2.9
**HIV/AIDS Stages**															
Asymptomatic	126	12.4		13.1**	1.8	14.4***	2.7	13.3***	3.6	11.7***	3.4	13.1*	2.9	14.8***	2.7
Symptomatic	508	50.0		12.6	2.3	13.2	2.8	12.3	3.4	11.4	3.2	12.5	2.6	13.8	2.8
AIDS	382	37.6		12.4	2.4	12.9	3.4	13.2	3.6	10.7	3.4	12.5	3.2	13.5	2.9
**CD4 cell count**															
< = 200	249	31.1		12.3**	2.4	12.8**	3.3	12.4*	3.5	11.2	3.3	12.5	3.0	13.6	2.9
200<cd4< = 350	249	31.1		12.8	2.1	13.3	2.8	12.4	3.7	11.3	3.5	12.6	2.8	14.0	2.8
350<cd4< = 500	194	24.2		12.9	1.9	13.7	2.8	13.1	3.4	11.3	3.1	12.5	2.8	14.0	2.7
>500	109	13.6		12.8	2.6	13.5	3.1	13.0	3.7	10.9	3.4	12.9	2.8	13.7	3.1
**Duration of ART**															
None	114	11.2		12.3***	2.2	13.3***	3.1	12.5	3.8	11.4	3.1	12.2***	3.0	13.6***	2.9
< = 1 year	196	19.3		12.0	2.5	12.4	3.3	12.2	3.3	10.7	3.3	12.0	2.9	13.2	2.9
1– < = 2 yrs	144	14.2		12.5	2.1	13.5	3.0	12.7	3.2	11.3	3.4	12.7	2.9	14.1	2.8
2– < = 4 yrs	270	26.6		13.0	2.3	13.6	3.0	12.9	3.7	11.3	3.4	12.8	2.9	14.0	2.9
4– < = 7 yrs	292	28.7		12.8	2.2	13.4	3.0	13.0	3.6	11.2	3.3	12.8	2.8	13.9	2.7
**History of drug use**															
No	548	53.9		12.6	2.3	13.3	3.2	12.6	3.5	11.1	3.3	12.5	3.1	13.8	2.9
Yes	468	46.1		12.6	2.3	13.1	3.0	12.9	3.5	11.3	3.3	12.7	2.7	13.7	2.8

ANOVA test; *** p<0.01, ** p<0.05, * p<0.1.

### Measurement Properties of the WHOQOL-HIV BREF

Table 2 shows the construct and reliability of the HRQOL measurement using the Vietnamese WHOQOL-HIV BREF. Six dimensions emerged from factor analysis that accounted for 60.0% of the variance. Three original domains, Physical, Spirituality, and Environment, remained in the reclassification of items. In addition, three new domains were constructed, namely Performance (individual performance of functional and cognitive activities), Morbidity, and Social. The first factor, Performance, accounted for 32.2% of the variance. The convergent validity of measure domains ranged from fair to good in correlation with the “Overall quality of life” and “Satisfaction with health status”, respectively. There were very small floor and ceiling effects. Cronbach’s alpha ranged from good to excellent across domains, ranging at (0.67; 0.89). As for criterion validity, the WHOQOL-HIV BREF could discriminate patients at different HIV/AIDS stages in Physical, Social, and Environment, and discriminate different CD4 cell count groups in Performance, Physical and Morbidity (Table 1). The instruments could also describe the changes in HRQOL of HIV/AIDS patients over the course of ART treatment in Performance, Morbidity, Spirituality, and Environment. Noticeably, HRQOL of patients who were taking ART more than 1 year was significantly higher than those in the first year ART; meanwhile there was a slight decrement in all measure domains in the first year of ART compared to those patients who were not yet treated.

**Table 2:** Factor loading, convergent validity and reliability of the WHOQOLHIV-BREF.

		Performance	Physical	Morbidity	Social	Spirituality	Environment
**Physical**	Pain and discomfort		0.63				
	Symptoms of HIV patients			0.65			
	Energy and fatigue		0.68				
	Sleep and rest	0.50					
**Psychological**	Positive feelings					0.74	
	Thinking, learning, memory andconcentration		0.52				
	Bodily image and appearance		0.46				
	Self-esteem	0.65					
	Negative feelings			0.41			
**Level of Independence**	Dependence on medicinal substancesand medical aids			0.41			
	Mobility						0.41
	Activities of daily living	**0.57**	0.54				
	Work Capacity	**0.59**	0.53				
**Social Relationships**	Social inclusion				0.58		
	Personal relationships	0.65					
	Sexual activity	0.59					
	Social support	0.51					
**Environment**	Physical safety and security						0.57
	Physical environnent (pollution/noise/traffic/climate)						0.78
	Financial resources				0.66		
	Opportunities for acquiring newinformation and skills				0.70		
	Participation in and opportunitiesfor recreation/leisure activities				0.74		
	Home environment	0.67					
	Health and social care: accessibilityand quality	0.74					
	Transport	0.66					
**Spirituality**	Spirituality/Religion/Personal beliefs					0.76	
	Forgiveness and Blame					0.75	
	Concerns about the Future			0.85			
	Death and Dying			0.79			
**Convergent validity**	Overall HRQOL	0.52	0.55	0.55	0.63	0.40	0.44
	Satisfaction with health status	0.67	0.59	0.54	0.60	0.41	0.47
**% floor**		0.0%	0.6%	0.6%	1.3%	0.7%	0.5%
**% ceiling**		1.4%	2.0%	1.2%	1.6%	1.9%	3.4%
**Reliability**	Cronbach's alpha	0.89	0.72	0.75	0.74	0.67	0.71

### Health-related Quality of Life Profile and its Associated Factors among HIV/AIDS Patients

Table 1 presents the HRQOL profile by various characteristics of HIV/AIDS patients. The average score was moderate across six domains; “Environment” had the highest score (13.8±2.8), and “Social” had the lowest score (11.2±3.3) (Table 2).

Table 3 shows the associations between patient’s characteristics and HRQOL domain scores. In the adjusted models, significant decrements were found across 6 dimensions of HRQOL in patients at provincial compared with those at central clinics. Socio-demographic characteristics of respondents were correlated with some HRQOL dimensions. Those patients who had higher education reported significantly higher scores in Performance, Environment, Physical and Social domains; and who had stable jobs reported in higher scores in Physical and Morbidity domains. Moreover, HIV/AIDS stage, immunological status, and the duration of ART remained in the reduced model in predicting HRQOL domains. Compared to asymptomatic patients, those at more advanced staged reported significant decreases in all HRQOL domains. The magnitude of differences was 0.2 0.6 times the standard deviation of the population means, corresponding to small to moderate decrements. Significant improvements in Physical and Morbidity were found in patients who had CD4 cell count of [350–500] compared to those had [< = 200] cells/mL. As for duration of ART, patients in the 1^st^ year ART reported significantly lower scores in Physical and Social compared to those who were not yet treated. Patients who took ART for more than 2 years reported moderate increments in Performance, Morbidity, Spirituality, and Environment.

**Table 3:** Factors associated with health-related quality of life among HIV/AIDS patients.

	Performance		Physical		Morbidity		Social		Spirituality		Environment	
	Coef	95% CI	Coef	95% CI	Coef	95% CI	Coef	95% CI	Coef	95% CI	Coef	95% CI
**Level of Health System** (^a^ Central)												
Provincial	−0.6***	(−1.0; −0.3)	−1.1***	(−1.5; −0.7)	−0.7**	(−1.2; −0.1)	−1.8***	(−2.5; −1.1)	−0.8***	(−1.3; −0.4)	−0.7***	(−1.2; −0.3)
District							−1.0***	(−1.7; −0.3)				
**Gender** (^a^ Male)												
Female							−0.6**	(−1.1; −0.1)	−0.4*	(−0.8; 0.0)	−0.3	(−0.7; 0.1)
**Marital status** (^a^ Single)												
Live with spouse/partner					0.4	(−0.2; 1.1)						
**Education** (^a^ Below high school)												
High school and above	0.5***	(0.2; 0.9)	0.6***	(0.2; 1.1)			1.0***	(0.5; 1.5)			0.6**	(0.1; 1.0)
**Employment** (^a^ Unemployment)												
Free lancer			0.5**	(0.0; 1.0)	0.7**	(0.0; 1.3)						
Stable jobs			0.6*	(−0.1; 1.2)	0.5	(−0.2; 1.2)						
Others							−0.7	(−1.5; 0.1)				
**Income quintiles** (^a^ Poorest)												
Poor					−0.6*	(−1.3; 0.0)			−0.7**	(−1.3; −0.1)	−0.7*	(−1.4; 0.0)
Medium									−0.6**	(−1.1; −0.0)	−0.5	(−1.2; 0.2)
Rich									−0.5*	(−1.1; 0.0)	−0.7**	(−1.3; −0.0)
Richest	0.3	(−0.1; 0.7)									−0.5	(−1.1; 0.2)
**HIV/AIDS Stages** (^a^ Asymptomatic)												
Symptomatic	−0.9***	(−1.4; −0.3)	−1.3***	(−2.0; −0.7)	−1.8***	(−2.7; −0.9)			−1.2***	(−1.9; −0.5)	−1.5***	(−2.2; −0.7)
AIDS	−1.0***	(−1.6; −0.5)	−1.5***	(−2.2; −0.8)	−0.9**	(−1.8; −0.0)	−0.6**	(−1.1; −0.1)	−1.2***	(−1.9; −0.5)	−1.8***	(−2.5; −1.1)
**CD4 cell count** (^a^ Less than 200)												
350<cd4< = 500			0.4**	(0.0; 0.8)	0.5**	(0.0; 1.0)						
**Duration of ART** (^a^ Not yet)												
< = 1 yr			−1.0***	(−1.6; −0.4)			−0.5*	(−1.2; 0.1)				
1– < = 2 yrs	0.3	(−0.2; 0.9)									0.7**	(0.0; 1.4)
2– < = 4 yrs	1.1***	(0.6; 1.5)	0.3	(−0.2; 0.8)	1.1***	(0.4; 1.7)			0.9***	(0.3; 1.4)	1.2***	(0.6; 1.8)
4– < = 7 yrs	0.9***	(0.4; 1.3)			1.3***	(0.6; 2.0)			1.1***	(0.5; 1.6)	1.0***	(0.4; 1.5)
**History of drug use** (^a^ No)												
Yes					0.4	(−0.2; 0.9)	0.3	(−0.2; 0.8)				
Constant	12.8***	(12.3; 13.4)	14.3***	(13.5; 15.0)	12.9***	(12.0; 13.8)	12.3***	(11.6; 13.1)	13.9***	(13.2; 14.5)	15.2***	(14.3; 16.0)

*** p<0.01,

** p<0.05,

* p<0.

## Discussion

We employed the WHOQOL-HIV BREF instrument in measuring HRQOL among HIV/AIDS patients in three epicenters of Vietnam. The findings contribute to the cumulative evidence on measurement properties of WHOQOL-HIV BREF in the Vietnamese settings. Using this measure, we found that HRQOL of HIV/AIDS patients were moderate across six measure domains; it was the lowest in Social and Performance and the highest in Environment. There were significant decreases in HRQOL of patients at lower levels of the health system. We determined various factors that influenced the HRQOL of patients with HIV/AIDS, including socioeconomic status (gender, education, employment, and income), HIV/AIDS stages, CD4 cell counts, and the duration of taking ART.

**Figure 1:**
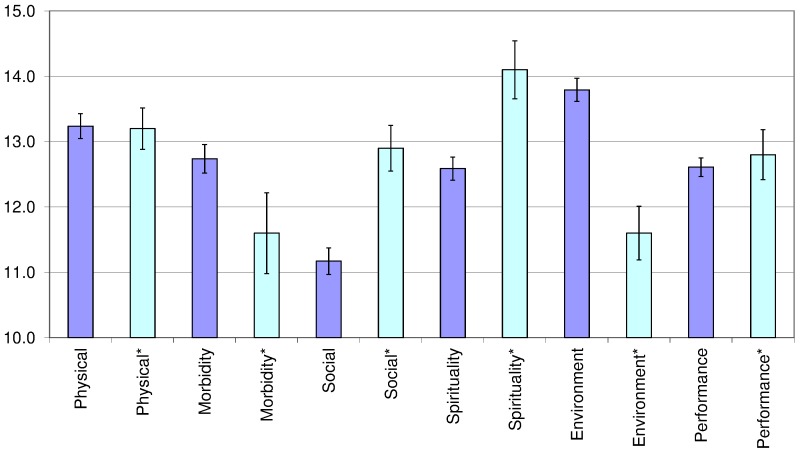
Comparison of means and 95% confident intervals of HRQOL domain scores in 2012 versus 2008 assessment.

This is the first assessment of HRQOL of HIV/AIDS patients taking ART across levels of the health system in Vietnam. It provides update information regarding the HRQOL outcome of ART services and its associated factors that is helpful for identifying additional interventions needed for patients during ART. In 2008, Tran *et al.* measured HRQOL in a convenient sample of 155 HIV/AIDS patients in one of the very first ART clinic in the northern Vietnam [5]. We compared findings of this present study with previous work in Figure 1, and found indifferent domain scores in Physical and Performance. However, there were significant increments in Morbidity and Environment, and decrements in Social and Spirituality in the 2012 assessment versus the 2008 study [5]. This could be explained by the remarkable efforts of the Government of Vietnam towards universal access to HIV/AIDS care and treatment for HIV/AIDS patients over the last few years. From 20% ART coverage for those patients in need of treatment in 2008, it has reached 50% by 2012 [30], [31]. Availability of free-of-charge ART services might encourage patients to seek health care service earlier and improve their compliance with treatment that in turn improve the HIV-related morbidity of patients. This study also found similar factors associated with HRQOL to previous works, for instance, employment was positively associated with Physical [4], [5]. However, we have not found that patients living with spouse or partner had poorer HRQOL than those who were single as did the 2008 study [5]. As for the duration of ART, there was a consistent finding that patients might experience HRQOL reduction during the first year of treatment [4], [5]. This might be due to the negative impact of side effects during early ART which was observed in Vietnam as well as other countries [32], [33]. Besides, we have found significant higher HRQOL among those patients who had taken ART for 2 years or more. Finally, the range of domains scores for asymptomatic (11.7; 14.8), symptomatic (11.4; 13.8) and AIDS (10.7; 13.5) patients in this sample were more varied and lower than that of the multi-center pilot study of the WHOQOL-HIV BREF, which were (12.9; 15;3), (12.1; 13.1), and (11.1; 12.0), accordingly [34].

The findings of measurement properties of the WHOQOL-HIV BREF may inform the selection of HRQOL measures in HIV/AIDS population. First, the range of internal consistency reliability of this measurement was [0.67; 0.89], similar to other studies [24], [35], [36], suggesting that the WHOQOL-HIV BREF could be used for measuring HRQOL at the population level. Second, our measurement shows that it has discriminative validity in different HIV/AIDS stages and immunological statuses. Moreover, this instrument was able to detect differences not only in Physical and Morbidity, but also in broader dimensions of HRQOL, such as Social, Environment, Spirituality, and Performance. Finally, we found the differences of HRQOL over ART periods that implies the potential use of WHOQOL-HIV BREF in evaluating the longitudinal impact of ART on HIV/AIDS patients. In fact, our previous work showed responsiveness of the generic WHOQOL-BREF in detecting clinically important changes in HIV/AIDS patients taking ART and methadone maintenance treatment [8]. Since this is a cross-sectional study, we are not able to confirm the responsiveness of the WHOQOL-HIV BREF; further longitudinal study would be necessary.

There are some policy implications derived from the study findings could be good references for the further expansion of ART services towards universal access targets in Vietnam. The decrements in HRQOL outcomes among patients at lower level of the health system raised concerns about the quality and efficiency of ART services at the grassroots level. In the rapid expansion of ART, services decentralization should go along with health care system strengthening in capacity, facilities, and cases management. In addition, gender-specific impact mitigation and support interventions should be in place. We found lower Spirituality and Social status in women that is similar to previous studies [5]. In Vietnam, women’s traditional gender roles as mothers and wives did not only made them not vulnerable to HIV/AIDS, but also confined them in some ways to living positively with HIV/AIDS [37]. Many women with HIV/AIDS were burdened by the responsibility of child rising. In addition, they were the ones who were taking care of their husbands among whom a significant proportion were drug users with complicated needs [38], [39]. Peer’s support, vocational training, job referrals, microfinance are potential interventions that have been proved effective in improving HRQOL and health status of women with HIV/AIDS [4], [38], [40]. Furthermore, it has been known in the Vietnamese setting that side effects and adherence difficulties as well as social stigma significantly influenced patients’ responses to ART [32]. The physical and social deterioration in the first year taking ART suggests additional attention and supports for patients when they begin ART.

The strengths of this study include a sufficient number of patients in three epicenters across levels of health systems and different regions of Vietnam. In addition, we employed an international instrument for HRQOL measurement which has been validated in the Vietnamese settings that enhanced the comparability of the study findings. Besides, there are some limitations. Fist, causal inference might be limited in a cross-sectional study design, particularly, for assessing the responsiveness of the WHOQOL-HIV BREF. In addition, patients at clinics were selected conveniently making the sample not representative for the population of HIV/AIDS patients. Therefore, generalizability of the results findings should be cautious.

In conclusion, the HRQOL outcomes of ART for HIV/AIDS patients were moderate across 6 measure domains. However, the efficiency of HIV/AIDS services at the grassroots level of health system should be improved. Gender-specific interventions, early treatment supports with focus on the first year of ART are recommended as integral interventions to improve the outcomes of ART services. Over the last 4 years, we observed significant improvements in morbidity but deterioration in social functioning of HIV/AIDS patients. The study highlights the important of regular assessment of HRQOL among HIV/AIDS patients in ART monitoring and evaluation.
